# Identification of gene mutation in patients with osteogenesis imperfect using high resolution melting analysis

**DOI:** 10.1038/srep13468

**Published:** 2015-08-26

**Authors:** Jianhai Wang, Xiuzhi Ren, Xue Bai, Tianke Zhang, Yi Wang, Keqiu Li, Guang Li

**Affiliations:** 1 Basic Medical College, Tianjin Medical University, Tianjin 300070, China; 2 Department of Orthopedic Surgery, Wuqing District People’s Hospital, Tianjin 301700, China; 3 Department of Medical Diagnoses, Tianjin Hospital, Tianjin 300211, China

## Abstract

Osteogenesis imperfecta (OI), a congenital bone disorder, is caused by mutations in *COL1A1* and *COL1A2* genes, leading to deficiency of type I collagen. The high resolution melting (HRM) analysis has been used for detecting mutations, polymorphisms and epigenetic alteration in double-stranded DNAs. This study was to evaluate the potential application of HRM analysis for identifying gene mutations in patients with OI. This study included four children with OI and their parents and fifty normal people as controls. Blood samples were collected for HRM analysis of PCR-amplified exons and flanking DNA sequences of *COL1A1* and *COL1A2* genes. Direct gene sequencing was performed to validate HRM-identified gene mutations. As compared to controls, HRM analysis of samples form children with OI showed abnormal melting curves in exons 11 and 33–34 of the *COL1A1* gene and exons 19 and 48 of the *COL1A2* gene, which indicates the presence of heterozygous mutations in *COL1A1* and *COL1A2* genes. In addition to two known mutations in the *COL1A2* gene, c.982G > A and c.3197G > T, sequencing analysis identified two novel mutations in the *COL1A1* gene, c.2321delC and c.768dupC mutations, which function as premature stop codons. These results support future studies of applying HRM analysis as a diagnostic approach for OI.

Osteogenesis imperfect (OI), also known as brittle bone disease, is a heterogeneous inherited disorder of bone. Patients with OI are born with defective connective tissue. The incidence of OI is approximately 1 in 10000 children^1^. According to clinical, pathological, and radiological criteria, OI phenotypes were originally classified into four subtypes (types I–IV) by Sillence^2^. Type I has mild deformity, with blue sclera and hearing loss, but dentinogenesis imperfecta is rare. Type II, the most severe form, is generally perinatal lethal. Type III is characterized by progressive deformity and is the most severe non-lethal type of OI. It is common for patients with Type III OI have hundreds of bone fracture. Type IV has moderate clinical phenotypes, with some symptoms similar to those in type I or type II^3^. Currently, based on the molecular genetic analysis of OI, including autosomal-dominant and autosomal-recessive patterns of inheritance and clinical symptoms, eight types (types V–XII) have been added to this disease.

The majority of types I–IV of OI are caused by autosomal dominant mutations in COL1A1 or COL1A2 genes, which encode the proa1 and proa2 chains of type I collagen^4^. Gene mutations in Type I collagen result in deficiency of collagens for connective tissue synthesis and defect of the structure of the assembled matrix collagen in the bone, tendon, ligament, and skin. All patients with OI have fragile bones, the most pronounced consequence of deficiency of type I collagen, Some of the symptoms that patients with OI may have are short body, loose joints, muscle weakness, sclera, curved spine, brittle teeth, and hearing loss^5^.

Currently, clinical examinations, including family and medical history and results from a physical exam, and radiological evidence provide major criteria for diagnosis of OI. However, these approaches have limitations for prenatal diagnosis and genetic counseling for OI. Detection of gene mutation has been applied for prenatal and postnatal diagnoses of OI. Since there are many mutations in *COL1A1/COL1A2* genes which are associated with OI and *COL1A1/COL1A2* genes have many exons, genetic sequencing for OI diagnosis is time consuming and the cost is expensive. Thus, studies to develop feasible genetic approaches for diagnosis of OI are needed.

High resolution melting (HRM) analysis is a recently developed genetic analysis method for fast, high-throughput post-PCR analyzing genetic mutations, such as single nucleotide polymorphisms (SNPs) and for identifying new genetic variants without sequencing. The method has many advantages, including high sensitivity, fast and easy operation, and low cost^6^. HRM analysis has been applied as a routine prescreening technique for many cancer predisposition genes and genetic diseases^7^.

The aim of this study was to evaluate the potential use of HRM analysis for identifying mutations in the *COL1A1/COL1A2* genes in patients with OI. We identified heterozygous mutations in *COL1A1* and *COL1A2* genes in children with OI using HRM analysis. These gene mutations were further confirmed by direct sequencing. Therefore, our results suggested that HRM analysis may serves as a potential method for OI diagnosis.

## Materials and Methods

### Study population

Methods used in this study were carried out in accordance with the approved guideline by the Committees for Ethical Review of Research involving Human Subjects at Tianjin Medical University. Informed consents were obtained from all subjects. This study included 50 normal people as controls, including 28 male and 22 female, with a mean age of 9-year-old. The physical examination results of these controls were normal. The controls had no relationship with any of these three patients. Family history, bone fracture, skeleton deformity and radiographic examination of patients were recorded.

**Figure 1:**
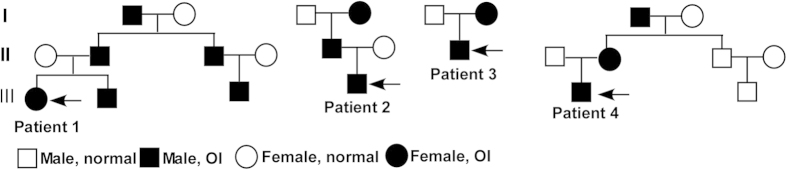
The pedigree chart of patients.

Four children diagnosed as OI were recruited to this study. The pedigree chart showed that all of these four patients have the portrait of the autosomal dominant inheritance (Fig. 1). None of them has consanguineous marriage in their families.

**Figure 2:**
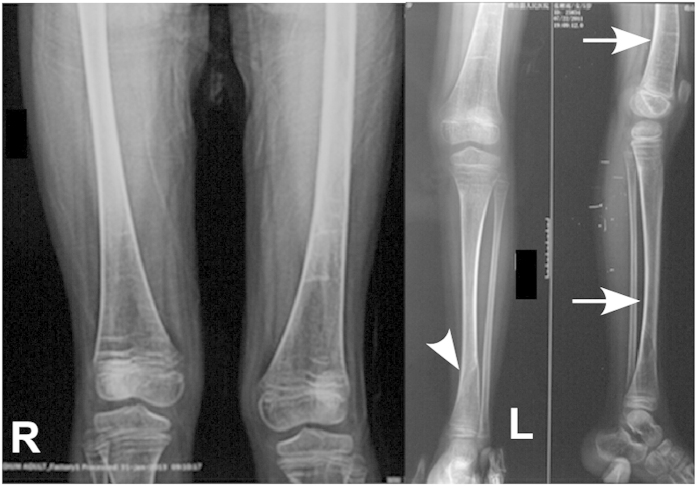
Radiographic evaluation for patient 1. Arrowhead: fracture in left femur. Arrow: deformity of left shinbone and femur.

Patient 1 was a 5-year-old girl (120 cm tall) with type I OI. She had blue sclera and normal teeth and walked normally. Her first fracture occurred at 2-year old, followed by 4 fractures in the four limbs. Radiographic evaluation showed meager bone cortex, low bone mineral density, and deformity of left shinbone and femur (Fig. 2). Her father, grandfather, younger brother and one cousin were diagnosed as type I OI. The hereditary mode of this family line was autosomal dominant inheritance.

Patient 2 was a 4-year-old boy (120 cm tall) with type I OI. He had blue sclera and normal teeth and walked normally. His first fractures occurred at 3-year old, followed by 4 fractures in the four limbs. Radiographic evaluation showed mild deformity of left shinbone, meager bone cortex, and low bone mineral density. The patient’s father and grandmother had bone fracture history, without OI diagnoses.

Patient 3 was 13-year-old boy (150 cm tall) with type IV OI. He had light blue sclera and brittle teeth. He walked normally. His first fracture occurred at 3-year old, followed by 3 fractures. Radiographic evaluation demonstrated severe osteopenia, deformity of the left femur, meager bone cortex, and low bone mineral density. Patient’s mother has dentinogenesis imperfect with fracture history.

Patient 4 was a 5-year-old boy (107 cm tall) with type I OI. He had blue sclera and brittle teeth. He walked normally. Her first fracture occurred at 2-year old, followed by 3 fractures in the four limbs. His mother had blue sclera and brittle teeth. His grandfather had blue sclera, brittle teeth, curved spine, and hearing loss. His mother and grandfather, without OI diagnoses, had no fracture history.

### Genomic DNA extraction

2 ml of peripheral blood was collected with EDTA anticoagulant. Genomic DNA was isolated using AxyPrep Blood genome kit (Axygen Scientific, Inc.), according to the manufacturer’s instruction. DNA quality and concentrations were assessed using NanoDrop 2000 UV-Vis Spectrophotometer (Thermo Scientific).

### PCR primer selection

Primers reported before for PCR amplification of COL1A1/COL1A2 genes with a size range of 80–260 bp (Supplemental Tables 1 and 2) were used. By using there primers, the entire *COL1A1/COL1A2* coding region and adjacent exon–intron junctional regions of *COL1A1/COL1A2* were amplified by PCR. For longer exon or exon regions with more complex melting domains, we designed primers (Supplemental Table 3) for PCR analysis. Primers were synthesized by Sangon Biotech (Shanghai) Co., Ltd.

### PCR amplification and HRM reaction

HRM analysis was performed using Type-it HRM PCR Kit (Qiagen). EvaGreen was used as Saturation dyes. Data were analyzed using illumina Eco fluorogenic Quantitative PCR System (Illumina, Inc.). Reaction conditions included an activation step at 95 °C for 5 minutes, followed by 40 cycles of 95 °C for 15 seconds, annealing conditions (58 ~ 65 °C) for 30 seconds, and 72 °C for 10 sec. PCR reactions were performed in a total reaction volume of 10 μl, containing 5 μl PCR Master Mix, 0.7 μmol/L of the forward and reverse primers, 3.6 μl template DNA and nuclease-free water, and 30 ng of genomic DNA. HRM was carried out over the range from 65 °C to 95 °C, rising at 0.1 °C/s, collecting 10 fluorescent signal data at each increment. Melting curve was analyzed using Eco V3.0 software. Each sample was performed duplicate.

### Amplification of mutated fragments for sequencing

All fragments showing HRM aberrant pattern were amplified by PCR analysis. The PCR products were sequenced at Sangon Biotech Co., Ltd. (Shanghai, China). Sequences from amplified COL1A1/COL1A2 were compared to know sequences using BLAST and GENETOOL. Mutations were searched in public databases, the GeneBank (number:NG_007400.1;NG_007405.1. To detect whether the mutation sites have been reported before, we searched osteogenesis imperfecta Mutation Database (https://www.le.ac.uk/genetics/collagen).

## Results and Discussion

### Genotyping by PCR-HRMA analysis

It is known that OI is associated with defects in type I collagen synthesis. Type I collagen consists of two proα1 and one proα2 subunits, encoded by *COL1A1* and *COL1A2* genes, respectively. *COL1A1* gene, containing 51 exons, is located on chromosome 17, and *COL1A2* gene, containing 52 exons, is on chromosome 7. For PCR-HRM analysis, we included 57 amplicons for COL1A1 and 64 amplicons for COL1A2 with a size range of 80–260 bp. We chose the optimal temperature and the primer concentration to generate specific products with efficient amplification and melting with an acceptable profile (Supplemental Tables 1 and 2). Melt temperature of each amplicon was checked using an online server to calculate thermal denaturation profile (http://www.biophys.uniduesseldorf.de/local/POLAND/poland.html).

**Figure 3:**
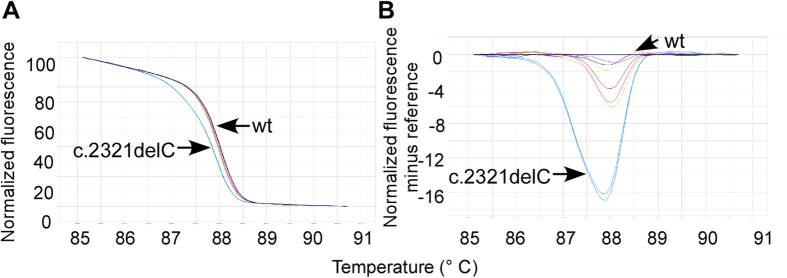
High resolution melting analysis of exon 33_34 in *COL1A1* gene. Melting curves are displayed normalized (**A**) and as difference curves (**B**). Normalized melting curves of the mutation, c.2321delC, as compared to the wild-type (wt) control.

Complete mutational screening was performed for samples from four OI patients and 50 normal controls. By comparing the melting curves of patients to those of controls, HRM analysis identified abnormal areas in the *COL1A1* gene with one mutation in exon 11 (patient 1, Fig. 3) and one mutation in exon 33–34 (patient 2), and in the COL1A2 gene with one mutation in exon 19 (patient 3A) and one mutation in exon 58 (patient 4). PCR-HRM screen *COL1A1/COL1A2* genes in normal controls showed no mutation. It should be noted that most single base variants can be genotyped by high resolution melting because many homozygotes differ in the primer melting temperature. However, the homozygotes of some single base variants, insertions and deletions have similar or identical melting temperatures and cannot be differentiated.

HRM software (Eco V3.0) was used to analyze normalized melt curve and difference melt curve for evaluation of mutation status in patients. Different shape of melt curve can be used to predict genotype change using PCR-HRM analysis^8^. The normalized curves provide the basic representation of the different genotypes, while difference plots show the difference between fluorescence of a patient’s sample and a control at each temperature transition. Heterotopous DNA double strands were unlocked because of DNA mismatch with temperature rising. Normalized melt curve and difference melt curve of all three patients were significantly different from those of control samples (Fig. 3B), suggesting that PCR-HRMA screening regions had variations and all three patient’s genotypes were heterozygous.

### Mutations identified by direct sequencing

**Figure 4:**
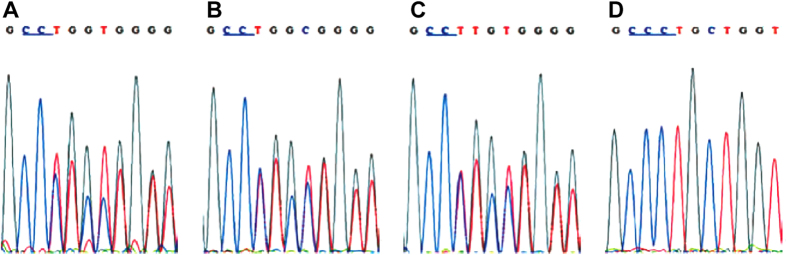
Sequences of exon 33_34 of *COL1A1* gene. (A): patient. (**B**): Patient’s father. (**C**). patient’s grandfather. (**D**). Normal control.

All pathological and nonpathological variants detected by HRMA were further analyzed by direct sequencing. The sequencing results identified mutations which were corresponded to abnormal gene regions detected by PCR-HRM screening. Two novel deletion mutations were found in patients 1 and 2. Patient 1’s sequencing result showed c.2321delC in *COL1A1* gene. This novel mutation was found in patient 1’s father and grandfather (Fig. 4). Another novel deletion mutation, c.768dupC, was identified in the exon 11 of *COL1A1* gene in patient 2. Patient 2’s father had the same mutation. Two known mutations were found in patients 3 and 4 by the sequence analysis. c.982G >A in the exon 19 of *COL1A2* gene was identified in patient 3 and patient 3’s mother. c.3197G >T in the exon 19 of *COL1A2* gene was found in patient 4 and patient 4’s mother and grandfather.

The c.2321delC mutation in *COL1A1* gene induces a translational frame shift (p.Gly774Leu fs*334). This mutation is predicted to generate a stop codon at 334 amino acids downstream of this site. The c.768dupC mutation in *COL1A1* gene leads to a translational frame shift mutant (p.Gly257Arg fs*30), which is predicted to result in a stop codon at 30 amino acids downstream of this site. Mmissense mutations in *COL1A2* gene were found in patients 3 c.982G >A, p.Gly328Ser) and patient 4 (c.3197G >T, p.Gly1066Val).

In our study, we found two novel mutations in *COL1A1* gene in OI patients, c.2321delC c.768dupC, which are not reported in the osteogenesis imperfecta Mutation Database, These two gene mutations result in premature termination codons, leading to degradation of mutant transcripts^3^ and reduced synthesis of normal collagen protein in OI. Glycine substitution of the helical region of *COL1A2* gene (c.982G >A, c.3197G >T) has been reported before, which is common in OI Type II–IV^9^,^10^. This evidence supports the relationship between the change of genotype and phenotype of OI.

HRM analysis for gene scanning is an attractive option for laboratories with time and resource constraints. In addition, PCR amplified products were analyzed by HRM, which reduces the risk of contamination. In our study, PCR-HRM analysis took 1.1 hours, with 5 minutes for HRM analysis. It should be noted that scanning accuracy depends on high quality PCR amplification. Thus, optimization of experimental condition is required for the success of HRM analysis.

Currently, HRM analysis has been used for genetic studies of tumors and potential diagnosis. Studies using HRM analysis showed that methylation of the BRCA1 promoter in peripheral blood DNA is associated with triple-negative and medullary breast cancer^11^. It has been reported that screening BRCA1 gene using HRM analysis was used for diagnosis of moroccan breast cancer^12^ and lung adenocarcinoma^13^.

In conclusion, our results confirm that the gene mutation detected by direct sequencing is consistent with the finding from PCR-HRM analysis. HRM analysis has a low cost, easy operation, fast, throughput and non-pollution advantages. Thus, high resolution melting is the method of choice for gene scanning of multiple exons for OI mutation screening. However, the number of case in this study is small, large scale trials are needed to evaluate the feasibility of HRM analysis for OI diagnosis.

## Additional Information

**How to cite this article**: Wang, J. *et al.* Identification of gene mutation in patients with osteogenesis imperfect using high resolution melting analysis. *Sci. Rep.* **5**, 13468; doi: 10.1038/srep13468 (2015).

## Supplementary Material

Supplementalm Tables 1-3
