# Supplementary material for: Identification of gene mutation in patients with osteogenesis imperfect using high resolution melting analysis
Source: Sci Rep. 2015 Aug 26;5:13468. doi: 10.1038/srep13468 (PMC4549685; doi:10.1038/srep13468)
Supplement: Supplementalm Tables 1-3 [file srep13468-s1.pdf]

Identification of gene mutation in patients with osteogenesis imperfect using high resolution melting analysis

Jianhai Wang[1#], Xiuzhi Ren[2#], Xue Bai[3], Tianke Zhang[1], Yi Wang[3], Keqiu Li[1], Guang Li[1*]

[1] Basic Medical College, Tianjin Medical University, Tianjin 300070, China

[2] Department of Orthopedic Surgery, Wuqing District People's Hospital, Tianjin 301700, China.

[3] Department of Medical Diagnoses, Tianjin Hospital, Tianjin 300211, China

# Jianhai Wang and Xiuzhi Ren contributed equally to this study.

[*]**Corresponding author:**
Guang Li
Professor
Basic Medical College
Tianjin Medical University
22 Qixiangtai Road, Heping District
Tianjin, P. R. China, 300070
Email: lig@tijmu.edu.cn
Phone: 011-86-22-83336839

Keywords: osteogenesis imperfect, high resolution melting, COL1A1/COL1A2, gene mutation

Supplemental Table 1. *COL1A1* primer sequences and qPCR-HRM amplification conditions

Exon	Forward Primer	Reverse Primer	Annealing Tem	Amplicon size (bp)
**1**	AGCAGACGGGAGTTTCTCCT	TCCTTGCACTCCCAAAAGTT	60°C	252
**2**	ACTGACAACGCCCCTCTTCCGCC	GAGCGCAGCCGCACCTGAG	60°C	240
**3**	GCC GAC GGG AGC AGC ATT AGC	AGAGGCCAGGTTAGAGAAGG	60°C	170
**4**	CCTCTTGTTTCTTCTCCCCC	CCACAGCCCAGAGTGCAACG	60°C	91
**5**	CGTTGCACTCTGGGCTGTGG	ACACACAAGGCCTCTCCACT	62°C	210
**6**	CTGCCTTTCAATTCACTGTCC	CTTCTGTCATCCATGCTCCC	60°C	154
**7**	ACAGAGGGATCACCATGACC	AGGAGGCATATGAAGACGTC	60°C	119
**8**	TGGAGAAGGTTGACAGGGACTTGTC	CCCAGGCCTGGGAGTTCTTCTATAG	57.5°C	186
**9**	CCACCTCTTCTATCTTTTCTAGGG	CCATGGGGTCAGATGGTATC	57.5°C	116
**10**	GGCATGATGGTCTTTTCTCTC	AGCTCCTAAATGAAGCCCAAG	60°C	143
**11**	TGTGTTCTAATGGCCCTTCC	GGGACTTGGGGAGCTTAAA	60°C	135
**12**	ACCCCCTCCACAGGTTCTTA	TGGAGGACCATGATGTTCAG	60°C	130
**13**	CAGAGTCCCACCATGAATGA	CTCTGGAACTGGGCACACTC	60°C	117
**14**	GAACAAGGCTGTCTCCCATC	CAAGGGGCCAGGAGTACTTA	60°C	125
**15**	AGTGGACTTAACGGGGCTTC	ACTGAGACCCCTCCCCACTC	60°C	131
**16§**	CTGCCATCTCTTCCTTCTCGCTG	CCATGAGGGTCATGCTTAGAGGAG	57.5°C	137
**17**	CCAGGGTGGCTTCTGATATG	ACACTGAGTCGGGGACACTT	60°C	159
**18**	GCCTGTTGCTGACACTCCTT	TGAAGCCTGGCAGGATACTT	60°C	101
**19**	CTCTGCTCCTAGGCTCACGCC	GTGGGCAGAAGGGAGAGTTTGGTAC	60°C	170
**20**	TCAGCTCATCCTCTTCTCCC	ATGGCGGGGAGACTTACAGG	60°C	100
**21**	CCACAGGCCGCCTCCTCCTC	GGG CAG GGA CAC TTA CAC GCT C	57.5°C	158
**22**	ACTGGCCTCCTCTCCTCCTG	CCGGCCGCAAGGAGAGGTTA	60°C	98
**23a**	AGCCACAAACTTGAGACCCT	TTCACCAGGAGATCCTTTGG	60°C	108
**23b**	CCAAAGGATCTCCTGGTGAA	TACACGGGATGGTCAGGGCC	60°C	112
**24**	CCTCTCTGCCTCCCTAGGGTCTG	GAGGCAGACAGGACAATGGCAGG	57.5°C	118
**25**	CACTGCTGCTTTCGTGCCTC	GGCTCTTCATGGATCCTCAC	60°C	160
**26**	CCTTTCTCCACAGGGAGAGC	ATGGGAAGGAGGTAGGGATG	60°C	103
**27**	TCCTAACCCTGAGTTCCCCT	AGGGTGTCTCCCCTTTTCTG	60°C	142
**28**	CTCGGCCTCCCCGTGACTGT	CTACCTCCCAGCATCCTGAC	60°C	128
**29**	GGGTAGGAAACACCTCTTTGGTCTC	CCT CTG GAT TCC CTG CAT CTC CC	60°C	180
**30**	GCTCACTGTCTGTTCCTCTCTCCC	CGCACCTTGACGGATGCAGC	60°C	116
**31**	CATGCCCTGTCCTTCCCTTCTAG	CCCTATCTCCATGGCTTTGGTCAT	60°C	226
**32**	CAGGCCTCACTCCAGTCTTC	AGATGGGAGCCATGTAGGG	60°C	190
**33_34***	CACTATAGGTCACCTCCTTGCCCC	CTGAGGAGGCTATGTGTTAGGGCA	60°C	215
**35**	CTGACCCTGAGCCTCTTGTCC	GGGCCAAGTATGGGGTCTTA	60°C	125
**36**	CAGCGCATCTCTCCAATCTGAC	GTCCCAGGTCCCAGTCGGTGATG	60°C	140
**37a**	AGTGGAAACTCCTGCCTCCT	GCCTTTAGCACCAGCATCAC	60°C	110
**37b**	GTGATGCTGGTGCTAAAGGC	GCATCAAGCCTGGACCCGT	60°C	141
**38**	ACGGGTCCAGGCTTGATGC	CAGAGAGGCGGGTGATACTC	60°C	113
**39**	CCCTCTGACTGTTCCTATGTTCTC	CAACCTAGAGCAGTGGACTCTG	60°C	123
**40**	TGACAGCCCCTCCTATCCTCATCC	CTGCAGAGATCTGAGCTGGCAC	60°C	214
**41**	GCTGCTCTCTGGCACCTCACCC	GCCCCACTCCAAGTCCTGTGATGG	60°C	209
**42a**	CCCTATCTCTGGCCTGACTCTTTC	CCACGTTCACCACTTGCTCCAGAG	60°C	82
**42b**	CTCTGGAGCAAGTGGTGAACGTGG	CTGCTCACCTCACGTCCAGATTCA	60°C	93
**43**	CATTGTGGCCTTGCCTAAGC	CTGTGGTCATGGAGTGTTGC	60°C	147
**44**	GACCCATATTCCCCTGCTCTCCC	CAGGGAACTGGAGCCCAGCTAC	60°C	163
**45§**	TCATGCTTTCCTTGCTCTCC	AAGGCATGGGGGACACAGCA	60°C	108
**46**	ACTTCCGCCTGACAGTTTGTCCC	GGGGAGGGGCTGAGCATACTTAC	60°C	168
**47**	CAGGCTACATTTGACGCTCACTG	GAGGAGAGAGAAGGCATGACTTAC	60°C	133
**48**	GCAGCCTTTCTCAAACCATT	GGGATGAGGGGCTACATACA	60°C	160
**49a**	CTCTTCCCTCTCTGTGCAGG	GAACCACATTGGCATCATCA	60°C	158
**49b**	CCGGGCTGATGATGCCAATGTGG	GAGGGAGAGGCTAGGGCAGGC	60°C	194
**50a**	TTTCCCTGACTCCATCTTGC	AGTTCTTCTGGGCCACACTG	60°C	155
**50b**	GGAGACTGGTGAGACCTGCGTG	CTCCTAACACTGGCTCTGAGGTCC	60°C	162
**51a**	CACTCTTCCCTCTCTCCCCT	ATGTAGGCCACGCTGTTCTT	60°C	154
**51b**	AAGAACAGCGTGGCCTACAT	GATTCTGGGCACTCACCGTG	60°C	154
**52**	CCCACCCCAGAGTCACACCGGAG	GAGCCAGGTTGGGATGGAGGGAG	60°C	181

* Alternate numbering 33-34 is based on the *COL1A2* standard.

§ Optimized primers concentration is 800 nM (where not specified, 400nM each primer is used).

Supplemental Table 2. *COL1A2* primer sequences and qPCR-HRM amplification conditions

Exon	Forward Primer	Reverse Primer	Annealing Tem	Amplicon size (bp)
**1a**	CACCACGGCAGCAGGAGGTTTCG	GCAGAGCCCCTGGGTCACCG	60°C	102
**1b**	CGGTGACCCAGGGGCTCTGC	GCTGAAGGCACTTACATTGGCATG	60°C	138
**2§**	TTGCTATTGATCCATGAAGTGATAC	GACATCACTGTAGCCAAGGGAATC	57,5°C	137
**3§**	GTATACTACACCAAAATGGAAGCTG	GAAGTAGTGTACTCTTACCTTTCTTAC	57.5°C	116
**4**	GTCCTGTTTGTATCTTTCCTGTAGGGC	AGTTATTCAAATACGGGACGAAGCCC	60°C	109
**5**	TACATAACAGGGTCCACCAGGCCC	CGTAAGACACCTTACCCCACCGAG	60°C	118
**6**	CTTCTCTAGAACTTTGCTGCTCAG	GGCTAAGATAAACAGATAAGCATACC	60°C	88
**7**	CACTGCTAAGTTGGTCATGTCTGAC	ACATAAGAGGCATTACAAGCTTTCAG	60°C	164
**8**	GCAGCTGGAGCCCCAGTAAG	CAGGACCCTAAGAAAATGGGAGACC	60°C	260
**9**	TGATGGGTCTCCCATTTTCTTAGGGT	GAGTTCTGTCAGGCATATTCAGCT	60°C	193
**10**	CCCCATTTTGTCTGATAGTTTACCAAG	TGTAAAAAGTCTCACCTGTGGTCCAA	60°C	129
**11§**	CTGTATTTCTTTCTAAGGGTGCTCG	AAGAATGTGCTCACCCTAATGCCT	57.5°C	82
**12**	GACCAAACACTATCATGGAACAGCATT	GAGGTCATGGGGAATTTCAATCAAG	60°C	163
**13**	ACTCAATCCTTCTCCATGTAGGGTG	GAATACAATGCTGAAGGATACAGTG	60°C	172
**14**	GTACAGGTTGGAAACTGAACAAAGC	GGAAGCACTTACAGCTGGGCC	60°C	157
**15**	GCTGTCATTTAAGTTTCCACCTGATC	GAAGACAGCACCCACCCCACA	60°C	152
**16§**	CCACCTTCTGCTTTGATTTCAGGG	CACCAGTGTTTTTACCTTGGGGCC	57.5°C	91
**17a§**	CCTTGGTTTAATTTCCTCTTTTAGGG	CTTACAGGAGGTCCAACGGGGC	57.5°C	128
**17b**	GCCCCGTTGGACCTCCTGTAAG	GAATATTTCGGTTCTTCTTTGACCC	60°C	121
**18§**	CCTTCTCCTTTCCTTTTCCTCATAG	GGTATACTCACAGCAGCACCCTTG	57.5°C	81
**19**	CAGCCCATCACCTCCCTAATGGAC	ATGATGAGAACCACAGTCATGACCAC	60°C	175
**20**	TGTTTGTCCTTTGACCACTGTTCTG	CCAGCTGGAGAAACTGGAATGAG	60°C	159
**21**	CATTAAACAGGGCTCTGCTGGGC	GAAGGGGTATCATAATCTGCCTCTGG	60°C	177
**22**	CTCTACCTTATCAAAGCCAAGAGATT	CCAGAAGCAGCAAGCCCCTTT	57.5°C	164
**23**	TTGAATAGGGCCCTCCTGGTAGTC	TGCCAGGTGTGATTTGCTCAGA	57.5°C	146
**24**	GGTGCCTTTGTTAGACTTCAGTTAATC	CTTGATAAGGGTGCAATAAGTGTCCT	60°C	198
**25**	CAGCATCATAAGCTTGAGGTTGTGAG	GTAAAATTTGGCTACAGAAAGTGCTG	60°C	222
**26**	GATTCAACATTGCAAAATCACCGTGG	GTTAGCACCTACCCGAGCACCA	60°C	151
**27**	TGGCTTGCAGCTAACCATCAGC	GTATGTCACCACTGTTGTCTAGCTG	60°C	212
**28**	TGCTCTCTTTCCTGTCACTTTCAGGGT	GGTGGAGAAGAGAGGTACGGTATGG	60°C	162
**28**	TGCTCTCTTTCCTGTCACTTTCAGGGT	GAGTTGACTTACCTGGAAGCCTGGAG	60°C	90
**29**	TCTCCTGAACGTAGCCATGGGA	GCATAGCAGTGGGGTATTAAACAG	60°C	182
**30**	TTCTCATGTTTTGTCTAGGGTCTCCAT	GGCTTTAAGGAGAAAGCACTACTAC	60°C	139
**31a**	GCTCGGAAGCTACACAAATGTAAAC	CCACTCTCACCTGGGGGACC	60°C	123
**31b**	GGTCCCCCAGGTGAGAGTGG	ACTTATTCCAGGCAGACTGGGCCA	60°C	145
**32**	GATTTCACATGTGTTTGACTCAAGGG	CCTTGTGCAGCCTTCTTTACTAG	60°C	192
**33**	TGCTCTTGCTTTATACTTTCAGGGTG	CAAACAAAATTCTACTCACACGAGC	60°C	95
**34**	GACAAGGTTCACTTTTGATGATACGGG	GCTGTTGGCTTAGTGAAAATGCATGC	60°C	148
**35**	CAGTTCTTTGAGCATCTATGTCAGGC	GGTTCTTACTTACAGGGCTTCCCCG	57,5°C	126
**36**	CTTCTCCCACCTAGGGTGAACGTG	GCTCTGGTATTCCGACCCACTCTAC	60°C	142
**37a**	GGTCCTGCTGTGAGTATCACATAATG	GGCCCTTTGGCTCCTCTTTCTCC	60°C	155
**37b**	GGAGAAAGAGGAGCCAAAGGGCC	AGCTGCTGTGTGGACCACCAGTG	60°C	116
**38**	ACAAGGGTTTGTTTGTGATTTGACTCC	AAAGCGGCGAGAGTCCATTGT	60°C	132
**39**	CCCATCTTACCCAAATTCTTGGAG	TCAGAAGCCAGGCCTTTTGGCT	60°C	177
**40a**	TGCTGCTCTCTTCCAGGCCCT	GGACCAGGAGGGCCAGAAATACC	57.5°C	88
**40b**	GGTATTTCTGGCCCTCCTGGTCC	GCCAGGGGGACCAACTGCACC	57.5°C	120
**40c**	GGTGACCAAGGTCCAGTTGGC	AGAGGAGTTGGAAATCACTTACAGC	60°C	121
**41**	TCTCTATTTAGGGACCTCCTGGCAC	CAGAATACTGTCAAGCACTCACCACAG	60°C	141
**42**	GGTTAGCATTCCATCGAATAAGGGG	GTTTCACACAATGAAATCCTGCTCAG	60°C	220
**43**	CATAGGGGCTGGTAGGCAGCAG	GAAGCTTAGCATCAATCTGGGTTGC	60°C	186
**44**	GCAACCCAGATTGATGCTAAGCTTC	CCACGAGAATGCTGCACTGAGGG	60°C	192
**45**	AGTGGGGAGGGGTATCTTGGGCC	CTTAACAGATGTTTTGGACTGATTCT	60°C	181
**46**	GCAGTATTTGTGGTGAAGTGAGTGC	CCTTCAGGATCAGTTTATGTGCGAG	60°C	225
**47**	TCTCTTGACATGTGCTCTGAAAGTGTG	CTGCTCGCTTTAGCCTCTATTTCAG	60°C	160
**48a**	GAGGCTAAAGCGAGCAGTGAGC	GCCTCGAATGCCAGCAGGTCC	60°C	171
**48b**	GGTCGCACTGGACATCCTGGTA	CCCCAAATCATACTTACAGCAGGGC	60°C	89
**49a**	GCTGCCATGGATGTCTCTCACTGTAA	GAGAAGGTGCTGAGCGAGGCTG	60°C	182
**49b**	CAGCCTCGCTCAGCACCTTCTC	AGAAGGGTCAGTCTGGTCTGGACA	60°C	201
**50a**	GAATCTGTGTTCTGCTCAATGAGAAG	GGGATGTTTTCAGGTTGGGCCCG	57.5°C	166
**50b**	CGGGCCCAACCTGAAAACATCCC	TGTGGGATTCCTCACCTGGCTGC	60°C	114
**51a**	ATCTGAGTCCTTCTCCACTTAACTGG	CTCCTCATCCATGTATGCAATGCTG	60°C	183
**51b**	CAGCATTGCATACATGGATGAGGAG	ACCCCCTCCATCCCACTTCCCA	60°C	185
**52a**	AGATTCAGAAATAGTGATGCTTTGTGT	AGGAAGGGCAGGCGTGATGGCTT	60°C	122
**52b**	AAGCCATCACGCCTGCCCTTCCT	GGCCAATGTCCACAAAGAATTCCTGG	62°C	82
**52c**	CCAGGAATTCTTTGTGGACATTGGCC	GAAGAAATGGAAGGATTCAGCTTTCAG	60°C	147

* This primers pair avoids the frequent intronic variant rs421587.

§ Optimized primers concentration is 800 nM (where not specified, 400nM each primer is used).

Suplemental Table 3. Primer sequences

Patient	Exon	Forward Primer	Reverse Primer	Amplicon size (bp)
1	COL1A1-33~34L	ATCCCGCTACACAAGTCAGG	AGTAGATGACCCCAGGAGAGC	489
2	COL1A1-11L	AACCTGACCTGCAACAATCC	GTCCACTCTCTGTCCCTTGG	468
3	COL1A2-19L	AGCAAACGGCCTTACTGGT	TGATGAAATGATGGCAGAGG	309
